# Closing the loop in person-centered care: patient experiences of a chronic kidney disease self-management intervention

**DOI:** 10.2147/PPA.S147831

**Published:** 2017-11-29

**Authors:** Kathryn Havas, Clint Douglas, Ann Bonner

**Affiliations:** 1 School of Nursing, Queensland University of Technology; 2 NHMRC Chronic Kidney Disease Centre for Research Excellence, University of Queensland; 3 Kidney Health Service, Metro North Hospital and Health Service, Brisbane, QLD, Australia

**Keywords:** patient-centered, content analysis, qualitative research, interviews, renal failure, self-care

## Abstract

**Purpose:**

The provision of self-management support (SMS) for people with earlier stages (1–4) of chronic kidney disease (CKD) can improve patient outcomes and extend time to dialysis. However, attempts to deliver such support have often not taken patient preferences into account. After the development, implementation, and quantitative evaluation of the person-centered CKD-SMS intervention, the aim of this study was to investigate participant experiences and perceptions of the program, as well as to seek suggestions to improve future SMS attempts.

**Patients and methods:**

Semi-structured, face-to-face interviews were conducted with almost all (63/66) participants in the CKD-SMS. Deductive categories were derived from previous research into self-management from the CKD patient’s perspective, and this was supplemented by categories that emerged inductively during multiple readings of interview transcripts. Content analysis was used to analyze interview data.

**Results:**

Participants recognized self-management of CKD as complex and multifaceted. They felt that the CKD-SMS helped them develop skills to engage in necessary self-management tasks, as well as their knowledge about their condition and confidence to take an active role in their healthcare. These participants experience a healthcare environment that is characterized by complexity and inconsistency, and participation in the intervention helped them to navigate it. The benefit of participating in this research to contribute to the scientific literature was also recognized by participants. Overall, participants found the CKD-SMS useful in its current format, and made some suggestions for future interventions.

**Conclusion:**

People with CKD must engage in self-management behavior within a complex health environment. Individualized SMS such as the CKD-SMS provides an opportunity to support patients to manage their health effectively.

## Introduction

For people living with chronic disease, daily routines often involve engagement in complex self-management tasks designed to slow disease progression and improve health outcomes. In chronic kidney disease (CKD), this involves a commitment to effective medication management as well as making lifestyle changes in areas including eating, drinking, physical activity, and smoking habits. Effective self-management from early in the disease process can improve important long-term outcomes such as time to dialysis and survival,^1^,^2^ and this is an ideal opportunity for self-management support (SMS) to be provided to this population. However, healthcare professionals (HCPs) and people living with chronic diseases have not always seen eye-to-eye with regard to how best to support self-management, or even what “effective self-management” means.^3^

Modern healthcare systems have developed over time to effectively deal with acute health conditions.^3^,^4^ This is problematic for individuals requiring chronic disease support, as acute and ongoing health problems require very different treatment. While provision of expert HCP care and instructions may be the most effective way to deal with an acute injury, ongoing management of long-term illness requires daily management by the person with the disease. Despite this necessary difference in treatment approaches, healthcare systems have struggled to adapt from a model in which HCPs are the sole experts and patients should simply comply with or adhere to instructions.^3^,^5^,^6^ In fact, while medical practitioners have long held “non-compliance” or “non-adherence” to be a failing on the part of a patient (or even a diagnosable condition in its own right),^3^,^7^,^8^ research shows that patients who do not follow HCP recommendations often have well thought-out, rational reasons for not doing so.^9^ In the broader chronic disease literature, there is a wealth of evidence that, while HCPs are the experts on health, disease, and treatments, people living with a chronic disease are experts on their bodies and their experience of what it is like to manage the disease on a day-to-day basis.^10^–^12^ People with CKD must be welcomed as partners in their healthcare and in driving research priorities and processes so that meaningful models of CKD self-management may be developed.^13^,^14^ A move in this direction will also lead to a deeper understanding of how HCPs themselves can be supported to provide person-centered SMS for individuals with chronic diseases. There is evidence that involvement of experienced chronic disease self-managers (“expert patients”^15^) in training of HCPs is useful, both for the HCPs and for their patients.^16^–^18^

**Figure 1:**
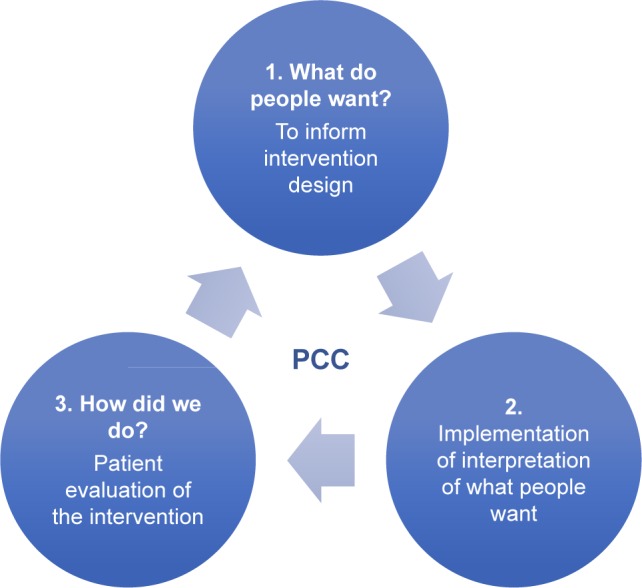
Closing the loop in person-centered research. **Abbreviation:** PCC, person-centered care.

Over the past three years, we have conducted a program of research which has aimed to keep the preferences and ideas of those with CKD, front and center, as the drivers of what SMS they need and how they would like to receive it to effectively manage their illness. We began by investigating preferences of people with CKD for receiving SMS,^19^,^20^ and used these findings (in conjunction with social–cognitive theory [SCT]^21^–^25^ and principles of person-centered care [summarized from published texts in Table 1]^26^–^29^) to develop and implement an SMS intervention for people with earlier stages of CKD – the CKD-SMS.^56^ This final phase is crucial in conducting person-centered research, as researchers cannot simply assume that they have been successful in achieving participant satisfaction and delivering what they set out to deliver. In achieving partnership with people with chronic disease, their feedback and advice with regard to research direction is of the utmost importance. The current study aimed to close the loop by investigating 1) participant experiences of the intervention, to establish whether we had succeeded in delivering person-centered SMS which was useful to those it had aimed to support, and 2) how future interventions might strive for improvement from the perspectives of individuals with CKD (see Figure 1).

## Material and methods

### Context and participants

To evaluate whether participants had found the CKD-SMS useful and investigate how it could be improved in future, participants in this study were those who had completed that intervention. The CKD-SMS was run in an individual format over a 12-week period for each participant. Every participant engaged in a (20–90 minutes) face-to-face session with the principal investigator (a researcher in CKD self-management who has a background in psychology), and then identified their preference for weekly, fortnightly, or monthly telephone sessions (which ranged from 5–60 minutes). At Week 12, all participants engaged in a second face-to-face session. All participants: had a diagnosis of stages 1–4 CKD; were aged ≥18 years; and were not suffering from distress or impairment which would hinder their participation (as determined by their treating clinician). To increase the trustworthiness of the study findings, it was intended for all who had completed the CKD-SMS to be invited to take part in the current study.^30^ Three individuals were unfit to participate due to health reasons; therefore, 63 of the 66 intervention participants were invited to take part in this study, and 100% of these people agreed to do so.

### Data collection

Data were collected by way of semi-structured, individual, face-to-face interviews with intervention participants, which were conducted 1 week after their final face-to-face session of the CKD-SMS. KH, the researcher who had delivered the intervention, conducted all interviews and, therefore, had built rapport with participants over the preceding 3 months. All participants were assured that their genuine feedback was appreciated, including constructive criticism of the program. Interview questions were developed with the goal of eliciting participant experiences and perceptions of the intervention,^30^ including: ways in which it had met and failed to meet hopes for SMS; effects of the intervention upon knowledge and confidence; and suggestions for future improvement. All interviews were conducted at the conclusion of follow-up quantitative data collection sessions. Interviews ranged from very brief (approximately 3 minutes) to a maximum of 30 minutes, and were conducted between July 2016 and March 2017. Interviews predominantly took place at participants’ homes or workplaces (86.4%), with the remainder taking place in public cafés or libraries, a renal clinic, or the researcher’s office. All interviews were audio recorded and subsequently transcribed verbatim for analysis.

### Data analysis

Content analysis was selected to analyze the data. As a model of SMS needs in CKD exists,^19^,^20^ a primarily deductive approach was employed, with supplementary inductive analysis to incorporate important new categories which emerged during the analysis. KH was the primary data analyst, and all categories and codes were discussed and agreed upon by all members of the research team (KH, CD, AB). Multiple examples of text from each code were discussed, and consensus was achieved regarding how data was coded. Elo and Kyngas’^31^ and Hsieh and Shannon’s^32^ approaches to deductive content analysis were used – the unit of analysis being individual interview transcripts. In the preparation phase, transcripts were read several times so that researchers could gain a firm understanding of their content. In the organizing phase, text was manually coded into a coding model. Deductive categories were formed based upon current understanding of areas important to people with CKD in receiving SMS.^19^,^20^ As researchers immersed themselves in interview transcripts, further meaningful categories emerged from the data inductively and were incorporated into the model. Content analysis is effective when conducted in this manner, as it is both based upon theory and existing research and allows for additional, important categories to emerge from the data.^33^ The research team then verified the codes within each category (see Table 2).^30^

### Ethical considerations

Approval to conduct this research was gained from the Royal Brisbane and Women’s Hospital (RBWH) and Queensland University of Technology (QUT) Human Research Ethics Committees (HRECs; EC00172 and EC00171; approval numbers: HREC/15/QRBW/500 and 1500001133, respectively). All participants provided written informed consent to participate.

## Results

Of the 63 participants, just over half (58.7%) were female, and age ranged from 25 to 84 years (M =56.9, SD =16.3). Approximately half (47.3%) had a grade 12 or lower level of education, and most were either retired (38.7%) or working full-time (33.3%). Approximately two-thirds of participants were married or in de facto relationships (64.0%), and the majority (69.3%) were born in Australia. Participants had been living with CKD for 4 months to 31 years, (M =94.7 months, SD =89.9 months).

**Figure 2:**
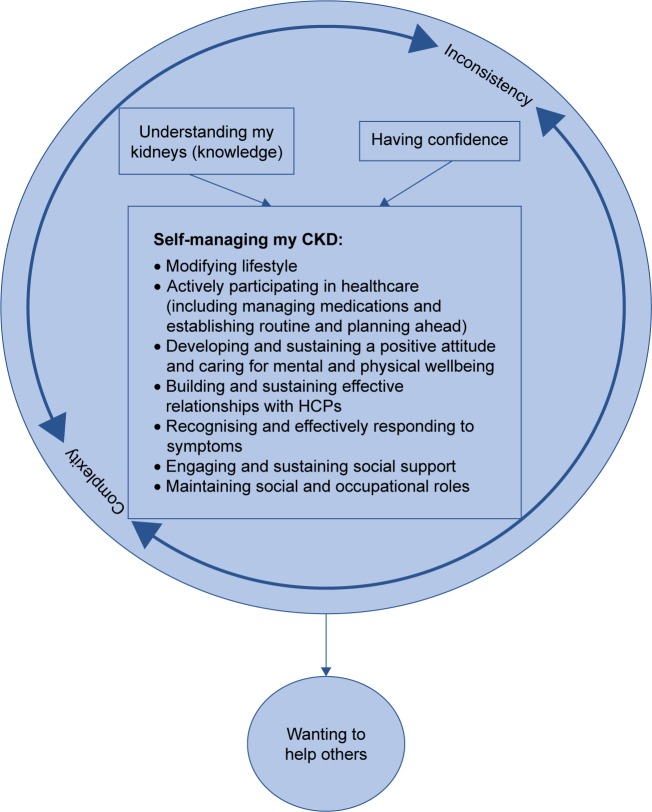
Model and context of self-management from the patient’s perspective. **Abbreviations:** CKD, chronic kidney disease; HCPs, healthcare professionals.

We conceptualized a framework of self-management in the context of the CKD-SMS in which “understanding my kidneys” and “having confidence” were central to “self-managing my CKD” (see Figure 2). Participants found the CKD-SMS helpful in providing support with aspects that previous research had identified as important to include in a self-management intervention for this population,^19^,^20^ and further areas were also apparent. Overall, it was clear that participants felt that the intervention had helped them to understand their kidneys and CKD (improved their knowledge) and had helped to build their confidence to manage their condition. Moreover, the CKD-SMS helped participants to develop a number of practical skills to engage in important CKD self-management behaviors. The intervention and its effects took place within an overall health environment fraught with complexity (of managing a chronic disease and, more often than not, multiple other health conditions) and inconsistency (in care plans, HCPs, and advice). All of this impacted the participants’ drive to help others (other people with CKD and/or to contribute to research), which some identified as a motive for intervention participation.

### Understanding my kidneys

The category “understanding my kidneys” captured five codes (or subcategories) which encompassed the beneficial effect of the intervention upon knowledge about kidneys and CKD. Participants recognized that the intervention had improved their knowledge, which many confessed was lacking when they commenced the CKD-SMS. For example, one participant reported “I’ve learnt more about the disease than I knew, which is a good thing.” Another, when asked what they had found helpful, stated “Just general information I suppose is most important … a better understanding of the workings of the kidneys.” At the conclusion of the intervention, participants felt they had a better understanding of how their kidneys worked, what their CKD meant, and what to expect should their kidney function worsen. Several participants were happy to have learnt how to understand their laboratory results, and felt that this helped them to better understand their HCPs and monitor their condition.

### Having confidence

The category “having confidence” included both confidence to engage in self-management behaviors and confidence in interactions with HCPs. Intervention participation built this confidence, with one participant saying “I’ve actually got a lot more confidence, and know that I can actually ask [my doctor] questions.” A different participant stated

It [the intervention] gives you confidence to carry on because you realize you’re doing just about all the right things anyway, that’s the main thing.

Participants felt reassured that they understood what they needed to be doing, and that they possessed the tools to self-manage their CKD.

### Self-managing my condition

Participants recognized engagement in self-management of CKD as being able to effectively complete seven practical, everyday tasks.

#### Modifying my lifestyle

A main benefit of the CKD-SMS consistently identified by participants was assistance with engaging in lifestyle modification. Depending upon the participants’ current lifestyle-modification understanding and success prior to entry into the intervention, the CKD-SMS helped participants to realize what they should be doing and to actually implement necessary changes. Participation enhanced understanding of the importance of making lifestyle changes, for example, one participant stated

I didn’t really realize … the importance of all the things that could help me improve my kidney function or kidney longevity type of thing … the exercise and fruit, and eating, you know. So that’s really helped.

Some reported that their participation spurred them to action, with one participant saying

… [the CKD-SMS] just gives you the little kick you need every now and then if you might be going off track, be it having too many drinks or you need to eat this certain amount of food and making sure you’re hydrated all the time.

#### Actively participating in my healthcare

After participating in the CKD-SMS, participants felt able to take a more active role in their healthcare. One reported this as

It’s made me more aware of self-monitoring … and even asking my GP what my creatinine is. And each time I have blood tests … I ask him what those two things [eGFR and creatinine] are, not just what the other blood results are.

Another simply said “I’m taking more responsibility, that’s for sure.” Active participation also encompassed more effective management of medications, with some participants identifying the benefits of tracking their regimens, for example “[I found it] good, putting in [tracking] your medication … really good.” Others were more aware of how to titrate their medications at home, and to manage over-the-counter medications, for example, “It’s [the CKD-SMS’] just given me a bit more to think about with regards to self-medicating and vitamins and whatnot.” People found some of the intervention resources (particularly handouts for tracking eGFR, blood pressure, physical activity, etc.) useful in helping them to get into good self-management routines. Some participants used these handouts as reminders/inspiration to commence engagement in routine self-monitoring behaviors, which can help people to identify and rectify problems quickly, before they worsen. One participant said “the little handouts that you gave to me to do my blood pressure and the daily planner [were particularly useful].”

#### Developing and sustaining a positive attitude and caring for my mental and physical wellbeing

Several participants found the intervention useful in terms of helping them manage their general wellbeing, especially with regard to psychological health. Stress was frequently identified as a problem, and participants found mindfulness and relaxation strategies that they learnt to be effective and able to be integrated into everyday life. One participant described that

I have now adopted … the leaves floating down the stream one [mindfulness exercise] … I use that to try and refocus so that I can get back to doing what I’m supposed to be doing.

The intervention and its resources led to participants feeling equipped with the tools that they need to manage stress in the future.

#### Building and sustaining effective relationships with HCPs

Many participants reported frustrations in their dealings with (often multiple) HCPs, and reported feeling that, in addition to boosting their confidence in their interactions with their HCPs, the intervention helped them develop practical skills to navigate these relationships more effectively. Participants felt less worried by the prospect of future appointments with their HCPs, and trusted their ability to get what they needed from these interactions. One participant identified feeling encouraged to persevere with HCPs until they address their concerns, saying “… I’ve been encouraged to keep asking even if I don’t receive the information in the first instance.”

#### Recognizing and effectively responding to my symptoms

Taking part in the CKD-SMS led participants to feel better equipped to recognize symptoms should they occur, as well as to take appropriate action (should that be necessary). This was explained by one participant as

Now … I know what to look for … the tiredness, being aware of how your body is feeling, the tiredness and the being dry and realizing that I’m not drinking enough, you know, just the general everyday things that go unnoticed until someone points it out to you.

Participants felt confident in the knowledge that they are the experts on their own bodies, and that they could identify and report changes as necessary.

#### Engaging and sustaining social support

Many participants were unaware of opportunities to engage with support systems for people with CKD (eg, online forums, support groups) before the CKD-SMS, and reported that their participation had made them realize that this support is indeed available. One participant stated “[I now] realize there’s a lot more support out there if I need it.” Moreover, people identified the usefulness of the intervention and its associated resources in helping them to engage with their existing support networks regarding their CKD-related needs. One participant discussed this as:

Part of living with a chronic disease is telling your support network about it so then they can support you … I was worrying about burdening them because they had busy lives … I think having the booklet [intervention companion handbook] just gives you a bit of an opportunity to explain to the people that you’re close to … about your illness … it’s good to have … all of the different aspects of kidney disease explained in one, simple, easy-to-read booklet.

#### Maintaining my social and occupational roles

Maintaining life roles in the face of chronic disease is challenging, and participants who completed the CKD-SMS recognized that they were provided with strategies to help them do so. One participant reported that she had received help with “… managing my expectations of myself, working full time with a chronic disease, particularly managing my fatigue levels.” Others identified how this particular intervention, in its flexibility with session timing and location, had allowed them to take part without any negative impact upon fulfilment of their social or occupational roles. For instance, a participant stated

It [flexible sessions] was really good for me, because I’m still at work and could play around with that also being able to see you at the weekend, that was a major help for me.

### Complexity and inconsistency

The healthcare environments of participants were characterized by complexity and inconsistency. They frequently had multiple comorbidities, and spoke of frustrations in managing multiple medical appointments through their week, and trying to reconcile contradictory recommendations and conflicting treatments from different treating specialists. Remembering large amounts of information given by multiple HCPs was also challenging. Participants disliked that they often saw different nephrologists each time they came to the clinic, finding it difficult to build rapport and extremely frustrating feeling that they were having to tell their story repeatedly. This was expressed by one participant as:

This [seeing a different nephrologist each time] is something I’ve really struggled with because my medical condition had become very complex … “when were you diagnosed? What treatment did you have?” Having to tell the whole story again, every time … this is so frustrating with doctors. They get a referral letter that tells your story but they don’t say “right, this is your story thus far.” They say “tell me your story” and I think “if I have to tell my story one more time!”

Many participants also identified the potential of self-management interventions such as this one to provide a consistent point of contact for them. One participant commented that:

It is a very complex area and … there are so many people involved in your care that it is really helpful to have somebody to help you navigate through all that … having an overall program coordinator … would be very helpful … it must be so easy to get lost in the system because you see all different medical specialists … and to try and keep that all tied together is really hard … the pharmacist, and the dietitian, and the psychologist, and the different medical specialists and then you’re having all these blood tests done and … who’s ordered them and who’s following up?

This difficult-to-navigate healthcare environment inevitably impacted upon the participants’ perceived understanding of their kidneys and their confidence in self-managing their CKD. For example, some participants had previously thought that they knew what they should do to take care of their kidneys, but had since received contradictory advice from a different specialist (to their nephrologist) who was treating them for another condition. Situations such as these led participants to doubt what they thought they knew, and damaged their confidence in engaging in self-management of their CKD. Unsurprisingly, given identified issues with inconsistency in HCPs, the vast majority of participants identified the importance of speaking with/seeing the same person each time when taking part in interventions such as this one. Recognition of how difficult navigation of these issues could be motivated participants to help others to do so if they were able.

### Wanting to help others

Lastly, for some who already felt quite confident in their self-management abilities, participation in this research was at least partly due to a desire to help others: other people with CKD; members of the research team; and the field of CKD research. Participants showed an understanding of the importance of improving healthcare for other people, with one reporting

If they ever wanted to do something [research], I’d jump at it, because I think any help we can give, you know, anything-advancements we can make with it….

It was also clear that people recognized that their participation in the person-centered CKD SMS was beneficial for everybody involved. For instance, one participant said

I always try to do these things [research projects] over the years of having my condition … it’s good for- to give them, like, knowledge, like my results … And it gives you guys training and helps me. It helps both ways, really … So I think it’s a very good thing. And I think there should be more of it. Because I’m willing to help out.

### Program feedback and suggestions

One of the main goals of this research was to investigate participants’ opinions regarding the process and format of the CKD-SMS. To achieve this aim, participants were asked several questions designed to elicit their opinions of how the program was run, with a view to using this information for refinement of protocols in the future. Every participant who was interviewed reported that they would recommend the intervention to a friend with CKD, indicating that they found it useful in its current form. In fact, most participants (71.4%) said that they would not recommend any changes to the intervention. Suggestions for changes to the CKD-SMS can be seen in Table 3. All participants (who were able to use it) reported finding the intervention companion handbook a useful resource, although fewer than half (44.4%) said that they had used additional handouts. Participants appreciated speaking with “someone who cares” and “understands” (ie, someone empathetic), and many (82.5%) identified the value of an individualized (person-centered), flexible format. On the other hand, two participants reported that they would have preferred a more structured approach (ie, one which covered set topics in set weeks with more of a lecture format). Only 22.2% of participants reported that they definitely would have traveled to an external location to participate in the intervention, while more than half (57.1%) said they could or would not have, and 20.6% were unsure. The benefit of having CKD-SMS as early as possible in the disease process was spontaneously mentioned by 20.6% participants, who expressed regret that they had not received this kind of support earlier. Only 14.3% of participants had a desire for group interaction in future CKD-SMS, and only one person (1.6%) thought that group sessions could be run instead of individual ones. Some (eight; 12.7%) participants wished for more face-to-face sessions within the CKD-SMS format, and a further five (7.9%) wished for ongoing support.

## Discussion

In this study, we have aimed to close the loop in providing truly person-centered SMS for people with CKD, by evaluating patient perceptions of the CKD-SMS intervention via brief, semi-structured interviews. This has been the final step in a person-centered research cycle which has involved: 1) reviewing the small amount of literature regarding SMS preferences of people with CKD;^19^ 2) directly assessing SMS desires of people with CKD;^20^ 3) attempting to deliver our understanding of the SMS that people with CKD wish to receive; and, now, 4) investigating whether we succeeded in delivering SMS that people with CKD found useful. In this final phase, we have gained support for our proposed model of CKD SMS from the patients’ perspective,^19^,^20^ as well as support for delivery of person-centered SMS. Consistent with our previous research, people with CKD recognized self-management of their condition as a complex, multifaceted process, and found that the CKD-SMS helped them to engage in this process more effectively. Failure to recognize the complexity and uniqueness of managing chronic disease on an individual level^5^,^6^ has been a consistent shortcoming of provision of SMS in chronic disease,^34^,^35^ as has the focus on biomedical markers rather than on goals that are personally meaningful to individuals.^3^,^36^ Participants in this study identified that, not only did participation in the CKD-SMS help them to practically engage in self-management tasks, it increased their knowledge about CKD as well as their confidence to take control of self-management of their disease.

The interviews we conducted provided some insight into the frustrations of trying to navigate the complexity and inconsistency of managing CKD. It is important to recognize that people with chronic diseases often exist in environments in which they are trying to reconcile conflicting advice from multiple HCPs, manage schedules involving numerous healthcare appointments, and take care of their CKD without causing problems for their other health conditions – all while trying to maintain their social and occupational roles. This is why a purely biomedical approach to chronic disease management is inappropriate,^5^,^34^–^43^ and why person-centered care – where patients are partners in their healthcare^44^ and experts on managing their condition in their individual circumstances^5^,^12^,^45^ – is crucial in achieving effective management of chronic diseases. It is helpful for patients to have a consistent point of contact who takes a person-centered approach (sees them and their overall condition holistically). In addition to recognizing the direct benefit of participating in the CKD-SMS, many participants felt a desire to help others, and understood the importance of contributing to research and advancing the field of self-management in CKD.

Findings from this study provide insight into logistical issues in delivering SMS for people with CKD, as well as ways to maximize feasibility of its implementation. Overall, participants would recommend the CKD-SMS in its current format, indicating that they found it a worthwhile intervention. While our previous research had indicated that most people would be happy to travel to a clinic in order to receive SMS,^20^ participants in this intervention recognized that the ability to engage in sessions at home/at a convenient location largely enabled them to participate. In fact, only about one-fifth of participants thought they would have been able to travel elsewhere to take part. The handbook given as part of the intervention was a useful resource, which is consistent with previous chronic disease research in which a desire for hardcopy materials was identified.^46^,^47^ However, loose handouts were mostly not used by participants in this study, and some reported losing these soon after receiving them. It may be important to include all materials in the one handbook resource, to minimize the chance that they will get lost and maximize their chance of being used. Only one participant in this study thought that SMS could potentially be run in a pure group format and, furthermore, identified difficulties of participants in traveling to receive support would be problematic for an attempt to deliver SMS in a group format in the future.

Consistent with previous chronic disease research,^48^,^49^ participants found the face-to-face interaction most valuable, and some desired more sessions and/or a longer intervention time period. Modification of the CKD-SMS protocol may lead to its ability to be delivered after renal appointments of patients in a pure face-to-face format, alleviating the need for phone sessions at all (one participant in this study believed they were superfluous even in this format). Patients regularly travel for these medical appointments anyway, minimizing the burden of additional travel to receive SMS. Delivering this support in conjunction with public sector medical appointments may also help to address identified issues with this support reaching the people who need it most.^50^ Current clinical practice guidelines recommend that earlier stages of CKD ought to be managed in primary care settings by general practitioners (GPs) and practice nurses for as long as possible, and implementation of support such as the CKD-SMS in conjunction with primary care appointments may mean that this is possible without compromising patient care. Furthermore, implementation of SMS in conjunction with primary care appointments may address participants’ identified desire for support earlier in the disease process, as participation could begin before kidney function declines enough for people to be referred to specialist renal services (often, when estimated glomerular filtration rate falls below 30 mL/min/1.73 m^2^).^51^ Some participants expressed a desire for specialized dietary intervention, despite the inclusion of dietary topics in the CKD-SMS and being encouraged to engage with dietitian services available through renal clinics. As such, there may be a role for interventions such as this, which focus intensively on things like meal plans and cooking classes, or GPs may be able to refer those who desire this type of support to appropriate services. People with other chronic diseases have also expressed interest in programs such as these.^52^ Interestingly, two participants reported feeling that the highly individualized format of the intervention was less beneficial than a highly structured, one-size-fits-all approach would have been. It is possible that these participants were at an earlier stage of activation (understanding, motivation, confidence, knowledge, and skills to self-manage)^53^,^54^ with regard to participation in their healthcare and, therefore, were not able to take as active a role in development of goals and plans for their time in the CKD-SMS. It may be important in future to include a measure of patient activation at the outset of interventions such as this one, so that the clinician has a better idea of the types of interactions that are likely to be beneficial for the patients.

This study was limited in that some interviews were very brief and, therefore, did not generate a lot of data. Furthermore, as the person who delivered the intervention conducted the interviews, there was some risk of social desirability bias in participant responses. It is not possible to claim generalizability in qualitative research, however; a strength of the study was that almost all (95.5%) of those who completed the CKD-SMS participated in interviews.

## Conclusion

People who participated in the CKD-SMS found it beneficial with regard to helping them to develop the knowledge, confidence, and skills to engage in necessary self-management tasks. This intervention may be modifiable for implementation in clinical practice by either a practice nurse in primary care settings or members of the multidisciplinary renal team in specialist renal services. It is crucial that SMS for people with CKD (and other chronic diseases) takes a person-centered approach where HCPs and patients work collaboratively to achieve meaningful goals.

## Figures and Tables

**Table 1:** Principles of person-centered care

Principle	Description
Respect for patients’ values, preferences, and expressed needs	Recognizing patient individuality and making an effort to deliver care which fits with their values, culture, and wishes.
Coordination and integration of care	Liaising with the patients other HCPs in order to ensure an integrated care experience in which patients receive complementary care and advice.
Information, communication, and education	Honest, transparent, easy-to-understand communication of health information and education regarding self-management behavior which may improve health.
Physical comfort	Alleviation of disease-related pain/discomfort and provision of a comfortable clinical environment.
Emotional support & alleviation of fear and anxiety	Recognition of the emotional impact of illness and efforts to respond to resulting issues such as anxiety and depression.
Involvement of family and friends	Accommodation of close loved ones in clinical appointments, correspondence, and decision-making and provision of support to those who take on a patient caregiver or support role.
Transition and continuity	Ensuring that patients and loved ones fully understand necessary regimens and are aware of warning signs to look out for, as well as pathways for follow-up.
Access to care	Provision of clear instructions regarding how patients may access care when they need it, and ensuring that processes (in hospitals, for specialist referrals, etc.) are as efficient as possible.

**Notes:** Data from Capuano et al^26^; Edgman-Levitan and Cleary^27^; Gerteis et al^28^; Shaller^29^; National Clinical Guideline Centre.^55^

**Abbreviation:** HCPs, healthcare professionals.

**Table 2:** Categorization matrix

Categories	Codes	Example
Understanding my kidneys	CKD and what to expect Kidney functions Meanings of laboratory results ESKD and dialysis Innovations in CKD treatment	Perhaps a little more knowledge of how to manage kidney disease and what I should be doing to enhance it, so that it doesn’t get worse … It’s made me more aware of what I needed to know and what I needed to focus on … So that’s been a good education.
Having confidence	To self-manage Interacting with HCPs	The main thing is I keep up my exercise and keep up my medications. I keep up my food, diet, and I just got to be more comfortable and self-confident that I can do it, and I feel – I feel that I am.
Self-managing my condition	Modifying my lifestyle	It’s made me think a bit more about what I should and shouldn’t be eating and doing … I’ve made a couple of little changes along the way which I guess has got to be a good thing.
	Actively participating in healthcare (including managing my medications and establishing routine and planning ahead) Developing and sustaining a positive attitude and caring for mental and physical wellbeing	The tracking sheets, they were the best, yeah. So, next time- and the appointment journals, yeah that was everything that I’ve never thought of doing myself. [the program has] had a big impact. I’ve been able to, to actually deal with stress better than I have been in the past. The family’s noticed that too. I’m not yelling at them as much … The stress relief guide is one of the best ones that I’ve seen for ages. I think it should be mainstream for everyone. Because it does, it does help because I was suffering with a bit of stress, so going through that, working through that as well, has done a lot of good and it’s alleviated some of the pain.
	Building and sustaining effective relationships with HCPs	I’ve actually got a lot more confidence and know that I can actually ask questions … And don’t have to think ‘oh the doctor’s going to tell me this’ and then come home and think ‘the doctor didn’t answer that question’. So being able to, to know that I can actually ask them questions.
	Recognizing and effectively responding to symptoms	Well I do understand now … that you can get anaemia from the kidney and I do understand that the kidneys can cause a lot more problems than what I even thought.
	Engaging and sustaining social support	Just to be able to know that there are, there is support for, for different types of kidney problems … there’s a lot more that can be done if- you’ve just got to ask.
	Maintaining social and occupational roles	[It has improved my confidence in] keeping up with family.
Complexity and inconsistency in healthcare	Appointment-management Contradictory advice from HCPs Remembering information across multiple illnesses Concern about conflicting treatments Seeing a different doctor each time	I think what it’s [the intervention’s] done is it does tie all of the different aspects around caring for my health together, because everybody – and particularly the health team – has a different role to play. The dietician only talks to you about what you’re eating. The pharmacist only talks to you about your medication and probably your GP is the closest person that comes to knowing your whole story, but it is helpful to have that all tied together and I’m sure that’s quite difficult to navigate for most people because it is very complex and the health professionals don’t always talk to each other. So, I think that anything that helps tie that all together for you is a positive.
Wanting to help others	Wanting to help further research Wanting to help others with CKD	The information I give you could save someone’s life next year, or even next week, so I don’t mind doing kidney research.

**Abbreviations:** CKD, chronic kidney disease; ESKD, end-stage kidney disease; HCPs, healthcare professionals; GP, general practitioner.

**Table 3:** Suggestions for future SMS interventions

Suggestion	Times identified (% of participants)
More face-to-face sessions	8 (12.7)
Integration with online/app interface	5 (7.9)
Specialized dietary intervention (ie, meal plans)	5 (7.9)
Ongoing support	5 (7.9)
Sessions after renal appointments at hospital	3 (4.8)
Structured, non-individualized approach	2 (3.2)
Involving HCPs	1 (1.6)
Enforced tracking/“homework”	1 (1.6)
Checklist for phone calls	1 (1.6)
Shorter program (6 weeks)	1 (1.6)
No phone sessions	1 (1.6)

**Abbreviations:** SMS, self-management support; HCPs, healthcare professionals.
